# Iliac vein aneurysms associated with May-Thurner anatomy

**DOI:** 10.1016/j.jvscit.2022.06.008

**Published:** 2022-07-08

**Authors:** Melissa K. Meghpara, Albertina Sebastian, Yi Tong, Alexander Shiferson, Robert Y. Rhee, Qinghua Pu

**Affiliations:** Division of Vascular Surgery, Maimonides Medical Center, Brooklyn, NY

**Keywords:** Iliac vein aneurysm, May-Thurner, Internal iliac vein

## Abstract

A 61-year-old woman with May-Thurner anatomy status post recent hysterectomy was found to have two iliac vein aneurysms on postoperative magnetic resonance imaging. Transfemoral venography showed the venous aneurysms received retrograde flow from the left internal iliac vein and the left common iliac vein (CIV) was compressed by the right common iliac artery. Both aneurysms were coil embolized and a left CIV stent was placed. Our initial experience suggests that iliac vein aneurysms may be caused by CIV compression and an endovascular approach is safe and effective to treat both lesions.

Iliac vein aneurysms are rare abnormalities and management is unclear owing to a paucity of study on this disease. The need for appropriate management is critical owing to the potential for deep venous thrombosis (DVT), pulmonary embolism (PE), or rupture. We describe our experience with endovascular treatment of rare iliac vein aneurysms associated with May-Thurner anatomy. The patient agreed to have case details published.

## Case report

A 61-year-old woman presented with incidental iliac vein aneurysms after total abdominal hysterectomy and bilateral salpingo-oophorectomy. She was otherwise asymptomatic and morbidly obese with a body mass index of 46. She denied a personal or family history of DVT, PE, or venous aneurysms.

**Fig 1:**
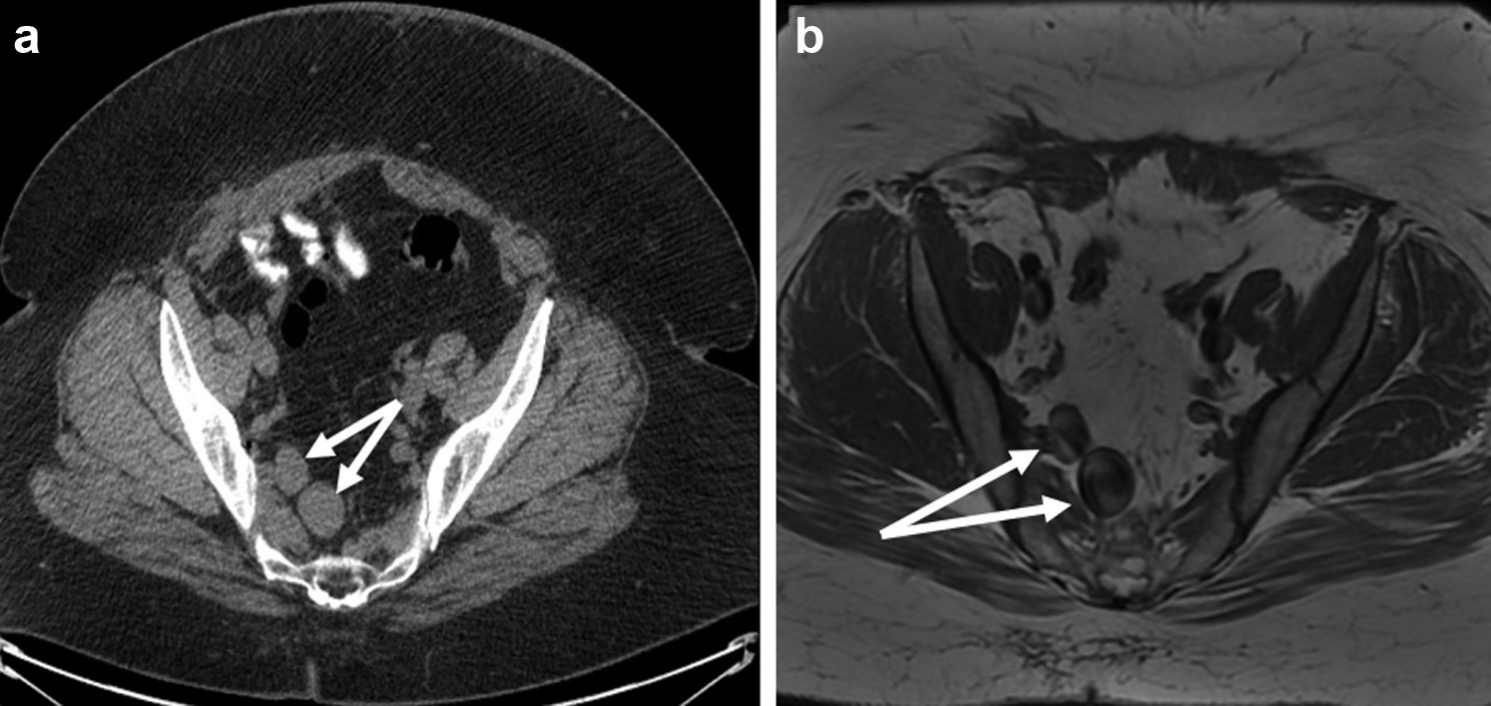
Computed tomography scan **(A)** and magnetic resonance imaging **(B)** demonstrating two right sided iliac vein aneurysms measuring approximately 2.5 and 3.0 cm with varicosities (arrows).

On computed tomography scanning and magnetic resonance imaging obtained by her gynecologist postoperatively, two right-sided iliac vein aneurysms were incidentally noted with varicosities (Fig 1). The aneurysms measured 3.0 cm and 2.5 cm separately. Venous duplex examination demonstrated no evidence of DVT in either lower extremity, the pelvis, or the abdomen. Given the size and risk of rupture or thromboembolism, the decision was made to treat both aneurysms endovascularly.

**Fig 2:**
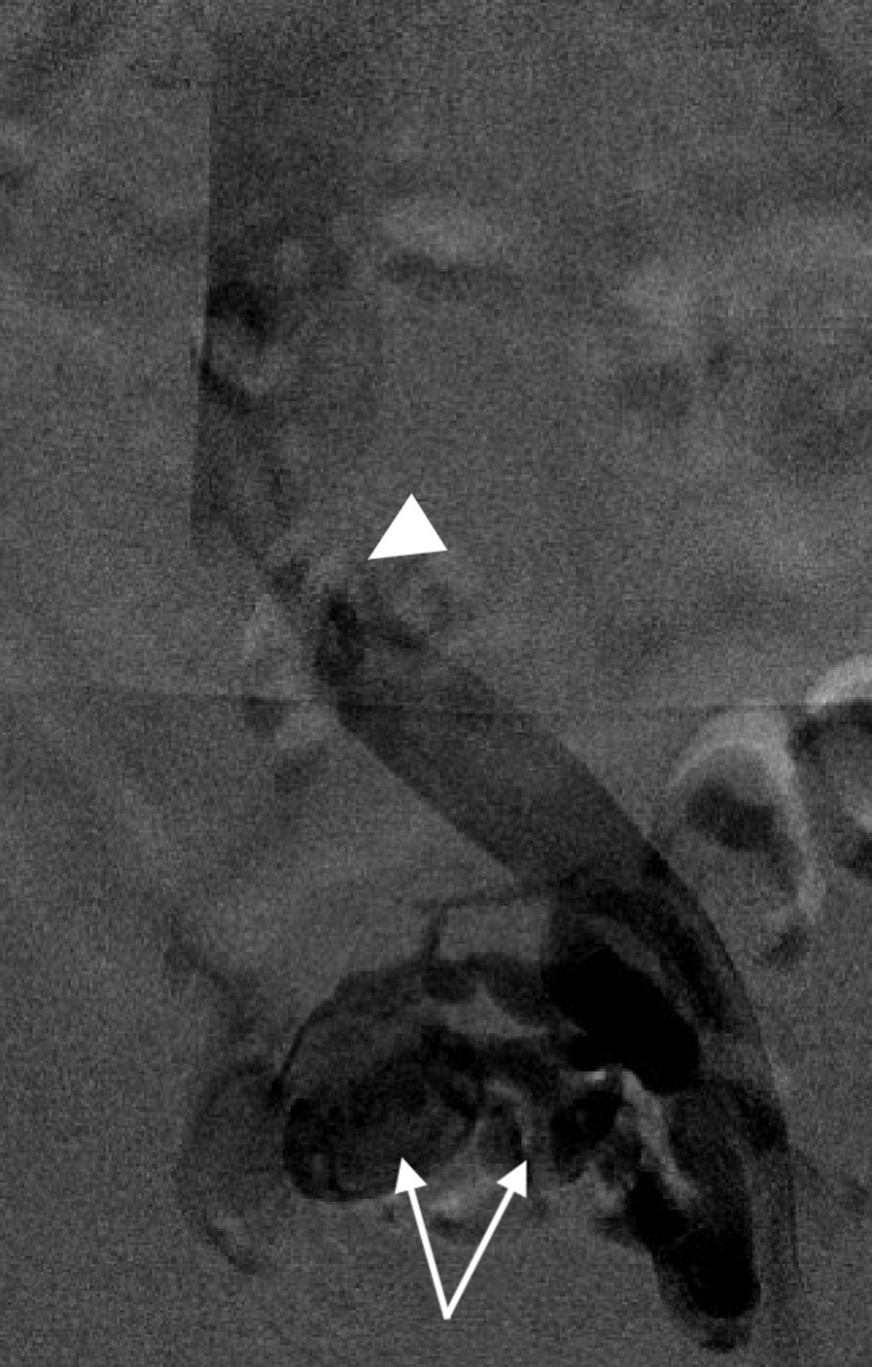
Initial venogram demonstrating two iliac vein aneurysms (arrows) with varicosities filling from the left internal iliac vein (IIV) and sign of left common iliac vein (CIV) compression (arrow head).

The patient was brought to the operating room and initial iliocaval venography was approached from the left femoral vein because the aneurysms were thought to originate from the right internal iliac vein (IIV). A patent left external iliac vein with a sign of compression of the left common iliac vein (CIV) was identified. There was reflux flow from the left CIV to the left IIV which lit up two venous aneurysms and emptied into the right IIV. Varicosities were also noted in the pelvis (Fig 2). There was no evidence of arteriovenous fistula (AVF) formation. We then navigated a 4F Glidecath across the inferior vena cava confluence and selectively catheterized the right IIV. A venogram demonstrated no reflux flow in the right iliac vein and no filling of the venous aneurysms. Based on these findings, we confirmed the diagnosis of iliac vein aneurysms and their association with venous congestion caused by May-Thurner anatomy. We decided to perform coil embolization of the aneurysms first and then stent the left CIV. The rational of the sequence is that stenting the CIV may decrease or stop retrograde flow to the iliac vein aneurysms, thus decreasing the visibility of aneurysms on the venogram, and deploying a stent in the left CIV may add difficulty to accessing the aneurysms for embolization.

**Fig 3:**
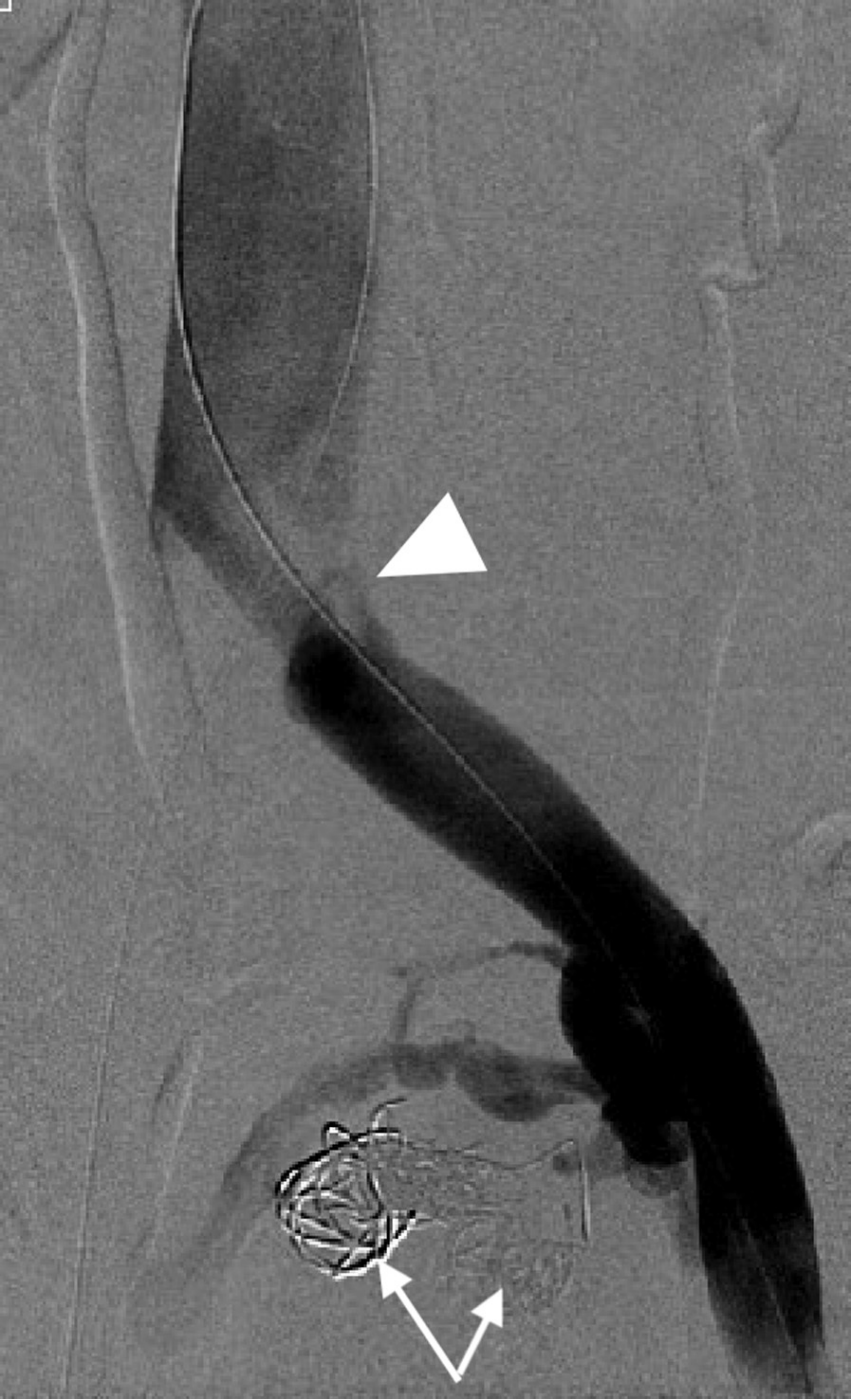
Venogram after coil embolization of iliac vein aneurysms (arrows) demonstrating thrombosis of the aneurysms and a sign of proximal left common iliac vein (CIV) compression (arrow head).

**Fig 4:**
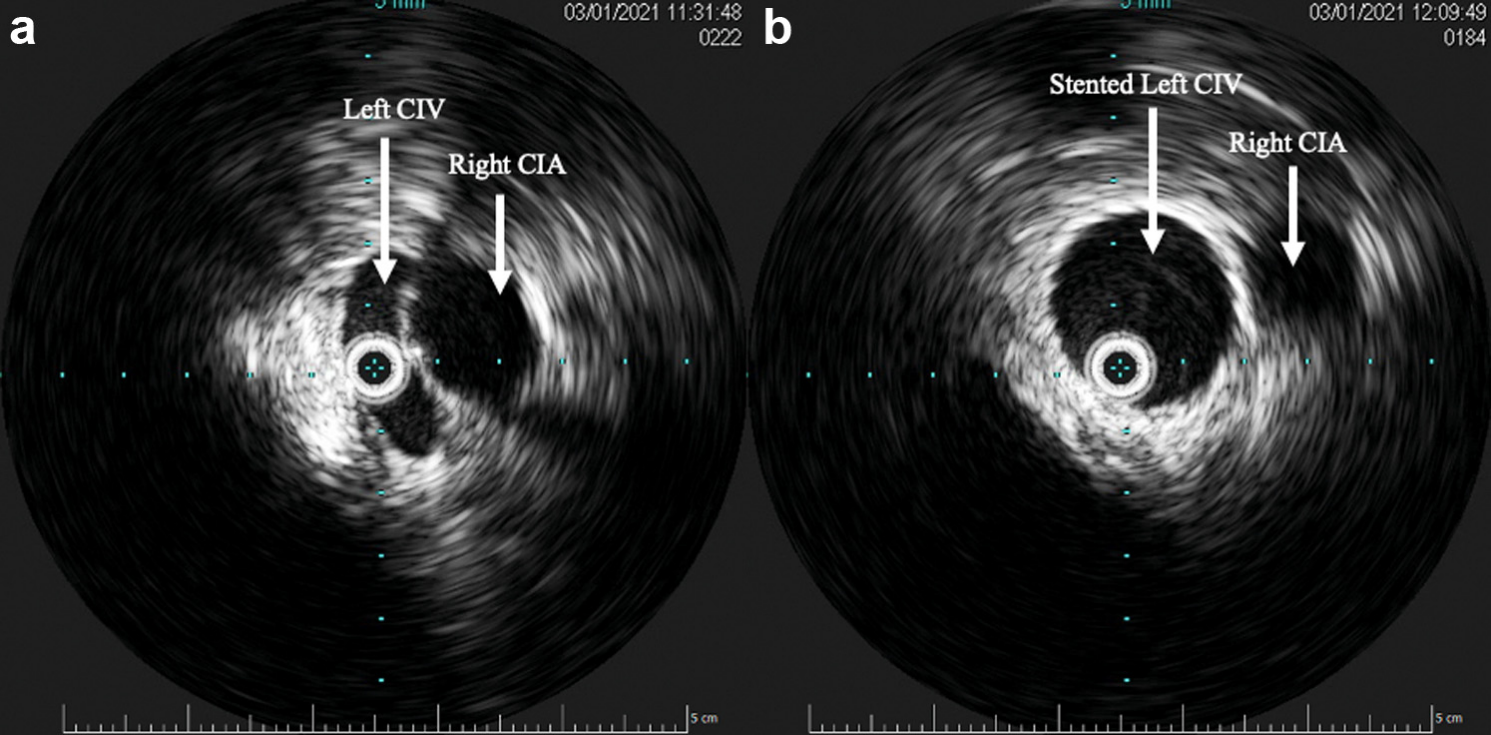
Preintervention intravascular ultrasound examination showing compression of the left common iliac vein (CIV) by the right common iliac artery **(A)** with resolution of compression after stenting **(B)**.

**Fig 5:**
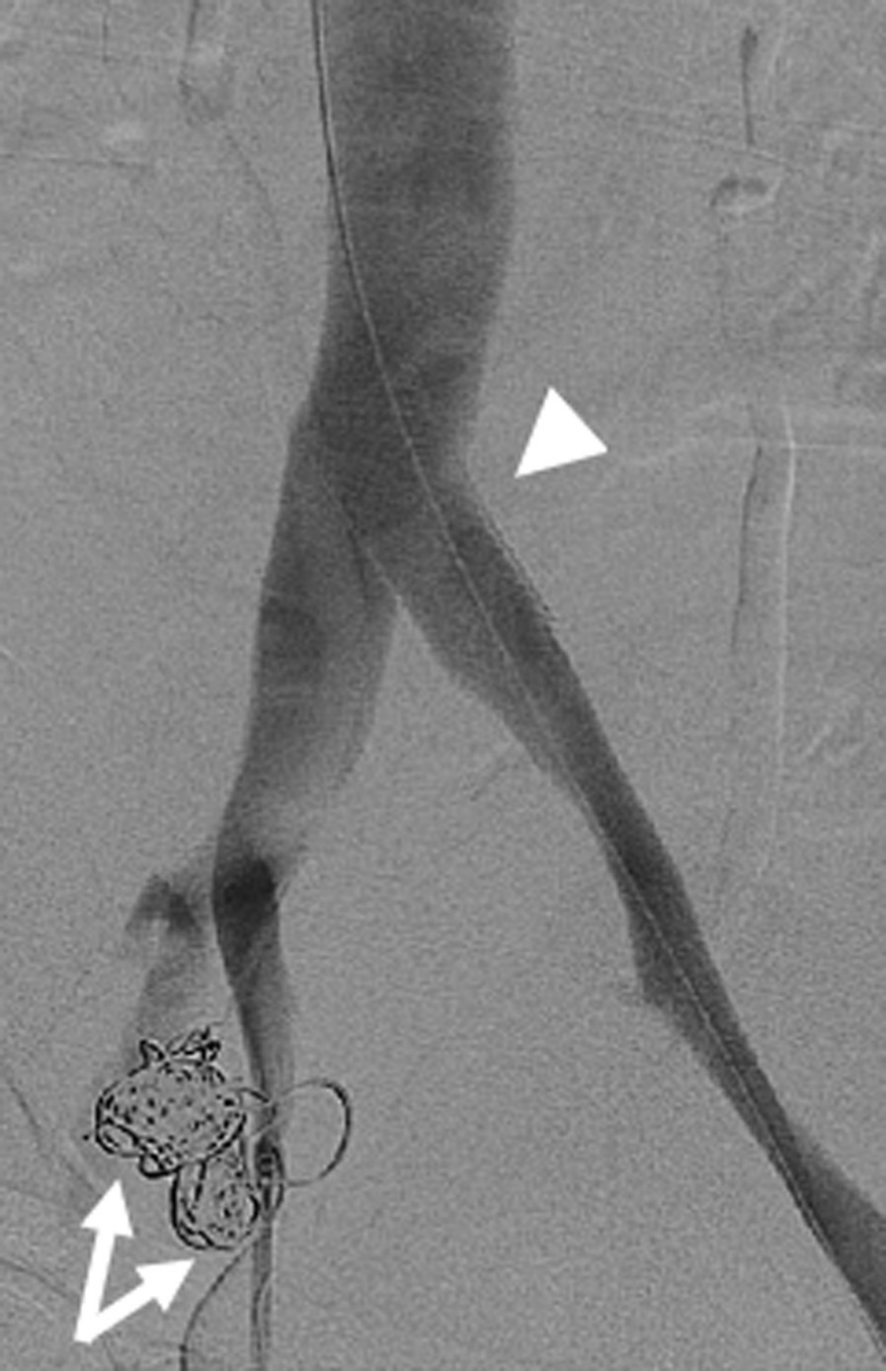
Completion venogram showing complete thrombosis of the venous aneurysms (arrows), lack of filling of varicosities, and resolution of proximal compression of the left common iliac vein (CIV) after stenting (arrow head).

Noting that the aneurysms originated from the left IIV, we accessed the right femoral vein and catheterized the left IIV from a contralateral approach; a 4F Glidecath was subsequently advanced into the aneurysms. Coil embolization was carried out on both aneurysms using 10- to 16-mm Nester embolization coils (Cook Medical LLC, Bloomington, IN) for the larger aneurysm, and 8- to 12-mm coils for the smaller aneurysm (Fig 3). A total of nine coils were deployed. After confirming thrombosis of the aneurysms, attention was turned to the left CIV compression. Intravascular ultrasound examination confirmed more than 80% compression of the left CIV by the right common iliac artery. The area of compression was angioplastied then stented with an 18 mm × 4 cm Venous Wallstent (Boston Scientific Corporation, Marlborough, MA). Because the left CIV compression was close to the iliac confluence (Figs 2 and 3), the stent was deployed 5 mm into the distal inferior vena cava from the left proximal CIV to gain adequate coverage of the lesion. Completion venogram and intravascular ultrasound examination demonstrated a patent left CIV with resolution of reflux flow and thrombosed venous aneurysms (Figs 4 and 5).

After the procedure, the patient was placed on aspirin 81 mg/d and apixaban (Eliquis) 2.5 mg twice a day for 3 months, then changed to only aspirin. She followed up at 2 weeks, 3 months, and 6 months without evidence of complications or recurrence and will now be followed yearly. Venous duplex examination at 2 weeks demonstrated a patent left CIV stent and no evidence of DVT or venous aneurysm. Magnetic resonance venography at 4 weeks showed a patent left CIV stent, thrombosed venous aneurysms, and no evidence of varicosities.

## Discussion

Unlike arterial aneurysms, venous aneurysms are rare, poorly studied entities that lack clear guidelines for management. They are classified as either primary or secondary based on etiology. Secondary causes include trauma, surgery, AVFs, infection, portal hypertension, or congenital abnormalities.^1^ Knowledge regarding these aneurysms largely rely on case reports and series in the literature. Teter et al^1^ assessed the anatomic location of venous aneurysms and found that it could be divided into four regions: head and neck, thoracic, intra-abdominal, and extremities, with popliteal venous aneurysms being the most common in the lower extremity. Zarrintan et al^2^ reviewed iliac vein aneurysms in 2019 and found only 50 cases with merely 4 internal iliac aneurysms. Significantly, the majority were left sided and external iliac aneurysms, with the most common etiology being AVFs.^2^ In our case, there was no evidence of an AVF on any preoperative or postoperative imaging studies and, given a finding of reflux flow in the left iliac vein with retrograde filling of the iliac aneurysms on venography, an iliac AVF was less likely because it usually enhances antegrade flow in iliac veins. Another causal factor should include May-Thurner anatomy, as was seen in our patient; a proximal left CIV obstruction by the right common iliac artery may lead to aneurysmal dilation of the pelvic venous system.^3^

Conservative management of aneurysms with anticoagulation to prevent thromboembolism has been proposed in the past; however, previous studies have demonstrated a greater than 60% risk of recurrent thromboembolism with anticoagulation alone.^4^ In addition to a concern for hemorrhage, AVFs, DVT, and PE with intra-abdominal and lower extremity venous aneurysms, chronic venous insufficiency with pain and swelling may also occur.^4^ Although it is rare, iliac vein aneurysm rupture has been documented in the literature.^5^^,^^6^ No specific size criteria or operative approach is recommended to manage venous aneurysm owing to a lack of evidence, but instead an individualized approach should be taken with contemplation of endovascular, open, and hybrid options.

Regarding intervention for iliac vein aneurysms, there is limited evidence for endovascular approaches. However, endovascular stenting and embolization have been used successfully, as in our case.^2^^,^^7^ Open repair with aneurysmectomy for iliac venous aneurysms can be useful in those with failed endovascular approaches or in the presence of an AVF.^2^^,^^8^ Still, others have opted to conservatively manage and surveil patients, with one patient being followed for 11 years without thromboembolic complications or aneurysmal growth.^9^

When contemplating the management of venous aneurysms, attention should be paid to several aspects of the aneurysm: location, size, symptomatology, and comorbid medical conditions. Based on the limited current literature, head, neck, and upper extremity aneurysms can generally be observed without significant concern for thromboembolism.^1^^,^^10^ Comparatively, intra-abdominal and lower extremity venous aneurysms have a higher risk of significant complications, including hemorrhage and thromboembolism. Increasing size and association with an AVF may also prompt intervention. Finally, although no single approach is advocated, it is reasonable to attempt endovascular treatment given advances in endovascular management techniques.

## Conclusions

In this case study, we found that asymptomatic iliac venous aneurysms are associated with May-Thurner anatomy and were able to treat the disease successfully with coil embolization and stenting. Given the rarity of cases in the literature, further studies are needed to confirm a causal association between May-Thurner anatomy and iliac venous aneurysms and to determine a standard management for the disease.
